# REDfold: accurate RNA secondary structure prediction using residual encoder-decoder network

**DOI:** 10.1186/s12859-023-05238-8

**Published:** 2023-03-28

**Authors:** Chun-Chi Chen, Yi-Ming Chan

**Affiliations:** 1 Department of Electrical Engineering, National Chiayi University, Chiayi, Taiwan; 2 MindtronicAI Co., Ltd., 7F, No. 218, Sec. 6, Roosevelt Rd., 24105 Taipei, Taiwan

**Keywords:** RNA secondary structure, Deep learning, Pseudoknot structure, Encoder-decoder network

## Abstract

**Background:**

As the RNA secondary structure is highly related to its stability and functions, the structure prediction is of great value to biological research. The traditional computational prediction for RNA secondary prediction is mainly based on the thermodynamic model with dynamic programming to find the optimal structure. However, the prediction performance based on the traditional approach is unsatisfactory for further research. Besides, the computational complexity of the structure prediction using dynamic programming is $O(N^3)$; it becomes $O(N^6)$ for RNA structure with pseudoknots, which is computationally impractical for large-scale analysis.

**Results:**

In this paper, we propose REDfold, a novel deep learning-based method for RNA secondary prediction. REDfold utilizes an encoder-decoder network based on CNN to learn the short and long range dependencies among the RNA sequence, and the network is further integrated with symmetric skip connections to efficiently propagate activation information across layers. Moreover, the network output is post-processed with constrained optimization to yield favorable predictions even for RNAs with pseudoknots. Experimental results based on the ncRNA database demonstrate that REDfold achieves better performance in terms of efficiency and accuracy, outperforming the contemporary state-of-the-art methods.

**Supplementary Information:**

The online version contains supplementary material available at 10.1186/s12859-023-05238-8.

## Background

**Fig. 1:**
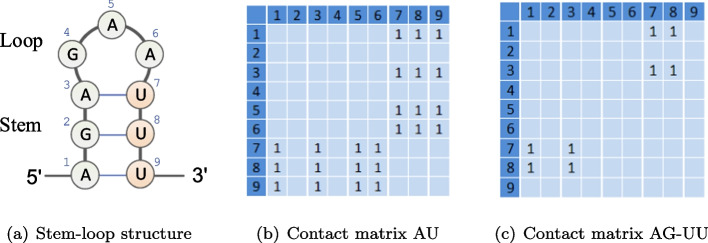
Illustration of the input conformations for the dinucleotide and tetranucleotide. **a** Example of the RNA structure with a stem-loop motif. The stem is the consecutive stacked base pairs and the loop is unpaired segments bounded by the base pairs. **b** The corresponding contact matrix of the dinucleotide AU. **c** The corresponding contact matrix of the tetranucleotide AG-UU

RNA is a single-stranded biopolymer with four types of nitrogenous bases (A, C, G, and U). It can have complicated structure motifs due to the local hydrogen-bonding interactions between the organic compounds. Studies have shown that noncoding RNAs (ncRNA) play important roles in cellular processes, including transcriptional regulation, chromosome replication, and interactions in processing RNAs and proteins [1–3]. Further efforts have been made toward the clinical applications of ncRNA in the diagnosis, prognosis, vaccine, and therapy [4, 5]. Besides, the RNA structure is found to be closely associated with its stability and functions, and hence RNA structure analysis is an important issue in biological research. To explore the mechanism of RNA function on a large-scale genomic database, computational prediction for RNA secondary structure is an efficient approach to analyze RNAs. In RNA, the secondary structure is to describe the hydrogen bonding interactions between the complementary base pairs. The canonical Watson-Crick base pairing includes AU and CG base pairs while wobble pair (GU base pair) is also frequently observed in RNA secondary structure [6, 7]. In most cases, the base-pairs appear in a nested style to form a stem structure (Fig. 1a), in which for any two base-pairs at the base positions $(i_1, i_2)$ and $(j_1, j_2)$ follows either $i_1<i_2<j_1<j_2$ or $i_1<j_1<j_2<i_2$. Another RNA folding motif is the pseudoknot structure, defined as a structure that contains non-nested crossing base pairs, and research shows that pseudoknots are recognized to play roles in structural stability and frameshifting function [8–10]. Nevertheless, RNA structure with pseudoknots makes it more challenging in computational RNA structure prediction. The conventional computational prediction for RNA secondary structure is based on thermodynamic models to find the minimum free energy through a dynamic programming (DP) approach [11, 12]. For example, Vienna RNAfold [13] and RNAstructure [14] are popular methods that use thermodynamic models to predict the secondary structure. However, the computational complexity of the RNA structure prediction using a DP algorithm for an RNA sequence of length N is $O(N^3)$, and finding the predicted lowest free energy structure including pseudoknots has a high complexity of $O(N^6)$ [15]. Besides, the prediction accuracy is limited by the quality of the tentative models.

Since parallel and distributed computing becomes widely accessible, deep learning methods can efficiently process large-scale data and make significant progress with remarkable performance. Consequently, deep learning has been extensively applied in a variety of fields, including biomedicine and bioinformatics as well. Due to the success of the deep learning, *CDPfold* [16] utilizes the convolutional neural network (CNN) to estimate the paired and unpaired probability. Based on the estimated probability, it then predicts the secondary structure through DP that improves the structure prediction for some RNA families without the pseudoknot motif. Further deep learning approaches try to integrate different learning models to enhance prediction performance. The long short-term memory (LSTM) network is able to learn the relationship between long-distance dependencies over the sequence, and *SPOTRNA* [17] uses multiple deep contextual learning models combined with LSTM to predict the base-pairing probability of the RNA structure. However, the LSTM model requires sequential processing with a large number of model parameters which makes it inefficient for RNA structure prediction. Instead of using recurrent models, *UFold* [18] adopts the U-Net model to capture the contextual information in the sequence that improves the accuracy of the RNA secondary structure prediction.

In this paper, we propose a new computational method called REDfold, which is based on the Residual Encoder-Decoder network to predict RNA secondary structure. Inspired by the advancement of *AlphaFold* [19] and *UFold* in the structure predictions, we utilizes encoder-decoder network following *FC-DenseNet* [20] to learn the local and long-range interactions among RNA sequence. We further incorporate it with the *ResNet* [21] network to avoid the gradient vanishing gradient problem by efficiently learning the residual information. By comparing our proposed algorithm REDfold with several well-known RNA secondary structure prediction algorithms, REDfold outperforms previous algorithms in terms of speed and accuracy. Additionally, We have developed a web server that allows users to easily predict RNA secondary structure through REDfold. The user can submit an RNA sequence to the server in FASTA format, and then check the predicted RNA structure.

## Methods

RNA secondary structure prediction aims to predict an accurate base-pairing structure of a given RNA sequence. In this work, we proposed a fast and accurate structure prediction algorithm that predicts RNA secondary structure through the deep neural network. The RNA sequence is first transformed into an input conformation consisting of contact matrices for the dinucleotide and tetranucleotide. After that, the encoder-decoder network can further extract the features and output a score map for the postprocessing. After the postprocessing, REDfold output the predicted contact map with the corresponding base-pairing structure, and the procedure is detailed in the following subsections.

### Preprocessing for input conformation

REDfold first converts the input RNA sequence into two-dimensional binary contact matrices as the input conformation. Similar to the protein structure prediction using contact maps to represent the interacting residue pairs, REDfold adopts the contact matrices to represent the relative positions of dinucleotide and tetranucleotide among the RNA sequence. Let RNA sequence $\underline{B}=(b_1,b_2,...,b_L)$ where each base $b_i\in \{A,C,G,U\}$ and *L* is the sequence length. The contact matrices for the dinucleotide $M(\underline{x})\in \{0,1\}^{L\times L}$, where the dinucleotide $\underline{x}\in \{A,C,G,U\}^2$, is to trace all 10 possible combinations of the base pairs $\underline{x}$ occurs in the sequence. Take Fig. 1b for example, the element $m_{ij}$ of the contact matrix *M*(*AU*) is one if the dinucleotide $(b_i\,b_j)$ belongs to the dinucleotide set $\{AU, UA\}$ without considering the base order. Using the non-ordered dinucleotide makes the prediction more robust to the RNA mutation that reorganizes bases while keeping the same secondary structure. Since RNA structures are related to consecutive dinucleotide (2-mer) contents [22, 23], the contact matrices for the tetranucleotide are to trace all 136 possible combinations of the 2-mer pairs in the sequence. The contact matrices for the tetranucleotide $\underline{y}$ is denoted as $M(\underline{y})\in \{0,1\}^{L\times L}$, where the tetranucleotide $\underline{y}\in \{A,C,G,U\}^4$. As illustrated in Fig. 1c, the element $m_{ij}$ of the contact matrix *M*(*AGUU*) is one if the 2-mer pair $(b_ib_{i+1}\,b_jb_{j+1})$ belongs to the tetranucleotide set $\{AG\,UU, UU\,AG\}$ without considering the 2-mer order. The last row or column in the contact matrix for the tetranucleotide is to trace the terminal bases of the sequence that can access the circular RNAs (circRNAs) as well. For instance, the element $m_{Lj}$ is to examine if the 2-mer pair $(b_Lb_1\,b_jb_{j+1})$ belongs to the combinations of the tetranucleotide $\underline{y}$. The input conformation thus consists of contact matrices $\textbf{M}$ with overall size $146\times L\times L$ for an input RNA sequence with length *L*. Based on the input conformation, the following neural network is able to extract the feature map and output a score map for the structure prediction.

### Network architecture

**Fig. 2:**
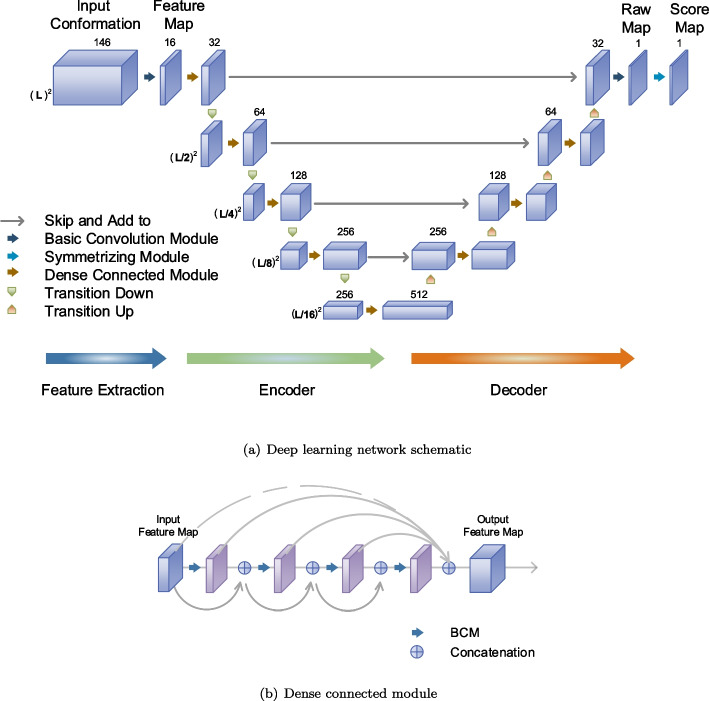
The REDfold architecture. **a** The learning network schematic, including feature extraction and encoder-decoder network. The RNA sequence is first transformed into an input conformation, and then fed into the deep neural network. Based on the extracted feature map, the encoder-decoder network outputs a score map for the secondary structure prediction. **b** Dense Connected Module (DCM). The DCM is a series of BCM layers and densely connected between layers. The output feature map concatenates all feature maps from the BCM layers and the output feature map in the encoder network includes the input feature map. Each layer receives all feature maps from the preceding layers to improve the network parameter efficiency

The deep neural network (DNN) of REDfold is composed of feature extraction and encoder-decoder network that is implemented based on the fusion design of *FC-DenseNet* and *ResNet*. As the input conformation consists of contact matrices with high sparsity, REDfold utilizes CNN with 3-layer basic convolution modules (BCMs) to extract the useful features for the RNA secondary structure prediction. The BCM is a basic processing unit that consists of 2-dimensional convolution, batch normalization, and rectified linear unit (ReLU). After the feature extraction network, the condensed feature map is of size $16\times L\times L$, and further fed into the following encoder-decoder network as shown in Fig. 2.

Since the feature maps closer to the input conformation are composed of low-level structure information, the encoder network in the DNN uses a hierarchical pyramid structure to extract the high-level structure features. In addition, the transition down module shrinks the size of the feature map by using down-sampling and BCM but increases the depth of the feature map with the dense connected module (DCM) to avoid forming bottlenecks in the encoding pathway. The DCM is a series of BCM layers and is densely connected between layers as illustrated in Fig. 2b. Each BCM layer in the DCM creates a new feature map and then it is concatenated with feature maps from all preceding layers before passing them on to the subsequent layer. Accordingly, the output feature map of DCM combines all feature maps including the input feature map that reuses all preceding features to reduce the number of network parameters. The DCMs can have more diversified features and improve the network parameter efficiency [24].

Next, the decoder network is composed of transition up and DCMs to reconstruct the spatial feature maps for the structure prediction based on the high-level encoded features. The transition up module utilizes up-sampling and BCM to expand the size of the feature map and decrease the depth of the feature map. Meanwhile, multi-level encoded features are introduced to the decoding pathway by adopting skip connection and direct summation as the residual connection in *ResNet* [21]. The reconstructed feature maps and the encoded feature maps with the same size are added directly with the skip and add to connection as shown in Fig. 2a. Compared to *FC-DenseNet*, the residual connection is able to learn the finer information in a more efficient way. Consequently, the decoder network generates a raw map with the size of $L\times L$ and passes it to the symmetrization to assure a symmetric matrix. At the symmetrization, the raw map is added by its transpose and subjected to the batch-normalization to reduce the internal covariate shift [25]. Finally, the network output a score map $\textbf{S}$ with the size of $L\times L$, and the element $s_{ij}$ of the score map represents the base-pairing score for the dinucleotide $(b_i,b_j)$.

### Postprocessing for structure prediction

In the final phase, postprocessing is required to make the predicted base pairs satisfy the following constraints for the RNA secondary structure.

1. The RNA base-pairing follows canonical Watson-Crick and wobble pairing rules.
2. The minimum length of the hairpin loop is at least 4 bases [26].
3. Each base cannot be paired with more than one base.

The problem of finding the base-pairing structure can be formulated as a constrained optimization similar to the approaches in the *Ufold* and *E2Efold* [18, 27]. In this optimization problem, the target is to find an RNA secondary structure that satisfies all the structure constraints and maximizes the overall base-pairing score. Assume $P\in \{0,1\}^{L\times L}$ is the predicted contact map with base-pairing structure corresponding to the input sequence $\underline{B}$, where the element $p_{ij}\in P$ is one if the dinucleotide $(b_i,b_j)\in \underline{B}$ form a base pair. To satisfy the first structure constraint, the contact map should follow the canonical and wobble rules that is $P\in M(AU)+M(CG)+M(GU)$, where *M* is the contact matrix considering a specific dinucleotide. Furthermore, the diagonally-striped element $y_{ij}$ should be marked out if $|i-j|<4$ to satisfy the second constraint. Hence, the optimization problem to find the structure satisfying all constraints can be formulated as follows.

$$\begin{aligned} \underset{P \in \Omega }{\textrm{maximize}}{} & {} \langle \textbf{S},P\rangle +\rho \,\Vert P \Vert \\ \text{ s.t. }{} & {} \frac{1}{2}(P+P^T)\textbf{1}\preceq \textbf{1}. \end{aligned}$$

where $\Omega$ is the sample space of all possible base-pairing structures satisfying the first two structure constraints, and the brackets $\langle \cdot ,\cdot \rangle$ denote matrix inner product. The hyperparameter $\rho$ is used to control the *L*1 regularization to improve the sparsity of the contact matrix. The last structure constraint can be dealt with through the inequality constraints in the optimization to limit at most one nonzero element in each row or column. Accordingly, the optimization criterion is to find the base-pairing structure satisfying the structure constraints as well as maximizing the similarity with the score map, and this constrained optimization problem can be solved efficiently by the primal-dual method [27–29]. Besides, the constrained optimization method can also work efficiently for the RNA structure with pseudoknots.

As REDfold utilizes the encoder-decoder structure with residual forward pass and constrained optimization technique, it is able to efficiently estimate the RNA secondary structure. The computational complexity of REDfold is $O(MN^2)$, where *N* is the sequence length and *M* is the parameters of the network. Furthermore, it can take advantage of parallel computing to accelerate the calculation and hence increase the overall throughput. Compared to the thermodynamic optimization methods that require time complexity $O(N^3)$ [30], REDfold is a highly efficient method for RNA secondary structure prediction.

## Results and discussion

In order to evaluate the performance of the proposed structure prediction method REDfold, *RNAStralign* [31] dataset consisting of 8 RNA families was used as the benchmark for performance assessment. As some sequences in 16 S_rRNA family are relatively long with respect to the majority of the dataset, the sequences with lengths over 720 bases were not included in the benchmark. Removing the outliers from the training data has been shown to avoid biasing the model in a neural network and it can also improve the memory efficiency to accelerate computing performance [32, 33]. Additionally, RNA sequences that contain unknown bases were excluded from the benchmark, and the constructed benchmark contains 24,315 RNA sequences in total. In addition to the *RNAStralign* dataset, we also took RNA sequences from the *Rfam* database 14.6 [34, 35] to construct the benchmark with diverse ncRNAs for further performance assessment. RNA families that contain over 120 members were selected in the benchmark, including 121 families in total. As a consequence, the constructed ncRNA benchmark consists of 39,517 RNA sequences, including 11,269 sequences with pseudoknot structure. The composition of the samples with respect to the specific family groups of ncRNA in the ncRNA benchmark is listed in Table S1 (Additional file 1).

We performed 4-fold cross-validation experiments based on the benchmarks to estimate the prediction accuracy. The benchmark was randomly divided into four folds of approximately the same size, and each fold was in turn taken as the test data for the validation while the remaining folds were taken as the training data. The ncRNA structure prediction performance was mainly assessed in terms of the accuracy (ACC) = ${(\text {TP}+\text {TN})}/{(\text {TP}+\text {TN}+\text {FP}+\text {FN})}$, the sensitivity (SEN) = $\frac{\text {TP}}{\text {TP}+\text {FN}}$, and the positive predictive value (PPV) = $\frac{\text {TP}}{\text {TP}+\text {FP}}$. The positive samples are defined as the bases in the sequence that form base pairs while the negative samples are the non-pairing bases. TP denotes the number of correctly identified positive samples, e.g., the bases $(b_i,b_j)$ are a base pair, and the pair position (*i*, *j*) is correctly predicted. TN denotes the number of unpaired bases (negative samples) that are correctly identified. FP denotes the number of negative samples falsely predicted as base pairs, while FN denotes the number of positive samples missed in the prediction. In addition to the base metrics, the harmonic metric F-score = $2/( \frac{1}{SEN}+ \frac{1}{PPV} )$ was also used for for performance evaluation.

**Table 1:** List of RNA structure prediction algorithms that were considered in this work for performance comparison

Program	Version/Package	Reference
*RNAfold*	Vienna 2.5	[13]
*RNAstructure*	RNAstructure 6.3	[14]
*Probknot*	RNAstructure 6.3	[36]
*CONTRAfold*	CONTRAfold 2.0.2	[37]
*SPOTRNA*	SPOTRNA (commit No. 6fb1c92)	[17]
*Ufold*	Ufold 1.2.0	[18]
*E2Efold*	E2Efold (commit No. f5d0aa7)	[27]
*MXfold2*	MXfold2 0.1.1	[38]

### Performance on RNAStralign

For comparison, several widely used RNA structure prediction algorithms with default configurations were evaluated on the same benchmarks, and Table 1 lists the algorithms considered in our performance evaluation. All machine learning-based methods were trained on the same training data for the evaluation except for *SPOTRNA* with no training module, and all experiments were performed on a 64-bit server machine running Linux kernel 5.8.0 with 8-core CPUs clocked at 3.5 GHz and 32 GB RAM. Table 2 summarizes the overall prediction performance and total run time (in seconds) based on *RNAStralign* dataset. Compared with the traditional algorithms based on thermodynamic models, the structure prediction based on deep learning can have manifest advantages in prediction accuracy. As shown in Table 2, REDfold yields highly accurate RNA secondary structure prediction results, outperforming previous structure prediction algorithms in terms of all accuracy metrics.

**Fig. 3:**
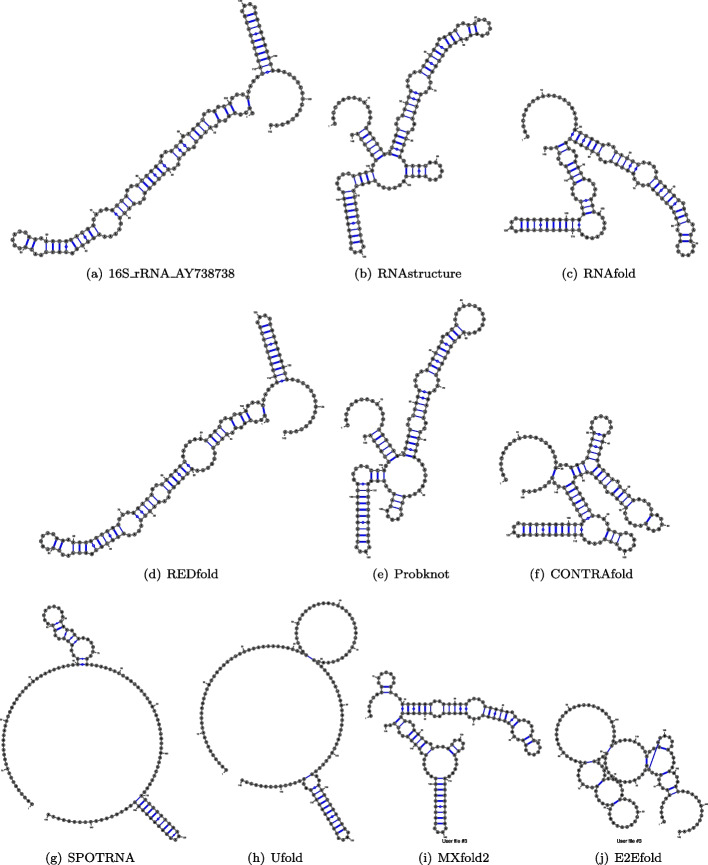
The illustration of the predicted secondary structures of 16 S_rRNA AY738738, which were drawn using VARNA [40]. The sequence length is 148 bases and the base pairs are connected with blue lines. **a** Native RNA secondary structure of 16 S rRNA AY378378. **b** RNAstructure (ACC=0.61, SEN=0.64, PPV=0.66). **c** RNAfold (ACC=0.69, SEN=0.71, PPV=0.73). **d** REDfold (ACC=0.92, SEN=0.91, PPV=0.95). **e** Probknot (ACC=0.59, SEN=0.67, PPV=0.64). **f** CONTRAfold (ACC=0.27, SEN=0.31, PPV=0.34). **g** SPOTRNA (ACC=0.58, SEN=0.33, PPV=0.82). **h** Ufold (ACC=0.54, SEN=0.24, PPV=0.83). **i** MXfold2 (ACC=0.65, SEN=0.67, PPV=0.70). **j** E2Efold (ACC=0.35, SEN=0.07, PPV=0.25)

Figure 3 illustrates the predicted secondary structures for 16 S rRNA (AY738738) from *RNAStralign* benchmark. Figure 3a shows the native RNA secondary structure and the predicted structure of REDfold as shown in Fig. 3d is able to make an accurate prediction. Besides, the accuracy of REDfold is high enough (ACC=0.92) such that the predicted structure was very close to the native one compared to other methods. For deep learning-based approaches, the deeper depth of a neural network is able to boost the capability for learning abstract characteristics. The depth of REDfold is up to 36 layers and the depth of Ufold is up to 19 layers; hence they can learn the critical features shared in RNAs and achieve higher accuracy compared to compact network models. In terms of prediction speed, REDfold is computationally efficient and the fastest algorithm within the methods with an accuracy higher than 0.7. To further evaluate the performance of the data with higher mutation diversity, the redundant sequences between the testing and training data are removed by using the program CD-HIT-EST[39] with sequence identify threshold 0.8. Table 3 summarizes the prediction performance with the redundant sequences removed and REDfold can still achieve high accuracy (ACC=0.895).

**Table 2:** Performance evaluation results based on the RNAStralign benchmark

	ACC	SEN	PPV	F-Score	Log$_{10}$(Time)
REDfold	**0.970**	**0.974**	**0.971**	**0.973**	3.515
*RNAfold*	0.519	0.636	0.554	0.592	**2.600**
*RNAstructure*	0.515	0.624	0.552	0.586	3.923
*Probknot*	0.536	0.632	0.571	0.600	4.260
*CONTRAfold*	0.613	0.708	0.632	0.668	3.499
*SPOTRNA*	0.703	0.739	0.725	0.732	5.589
*Ufold*	0.950	0.966	0.944	0.955	5.023
*E2Efold*	0.671	0.632	0.734	0.679	4.984
*MXfold2*	0.862	0.892	0.819	0.877	4.157

The measures ACC, SEN, PPV, and F-Score are utilized as the accuracy evaluation, and the computation time was measured for completing the structure prediction of the entire benchmark (in seconds)

Top-performing items are emphasized with bold font

**Table 3:** Performance evaluation results based on the RNAStralign benchmark with the redundant sequences removed

	ACC	SEN	PPV	F-Score	Log$_{10}$(Time)
REDfold	**0.895**	**0.905**	**0.906**	**0.906**	2.126
*RNAfold*	0.496	0.617	0.540	0.576	**1.763**
*RNAstructure*	0.494	0.607	0.539	0.571	3.089
*Probknot*	0.510	0.616	0.553	0.583	3.427
*CONTRAfold*	0.569	0.663	0.601	0.631	2.555
*SPOTRNA*	0.648	0.628	0.705	0.664	4.650
*Ufold*	0.835	0.862	0.844	0.853	4.038
*E2Efold*	0.477	0.343	0.545	0.421	3.805
*MXfold2*	0.701	0.747	0.723	0.735	3.204

Top-performing items are emphasized with bold font

### Performance on the ncRNA benchmark

For the sake of evaluating the effectiveness of REDfold for more various ncRNAs, we used the ncRNA benchmark constructed from the *Rfam* database to estimate the prediction accuracy. Table 4 summarizes the structure prediction results based on the ncRNA benchmark, and REDfold can have better prediction performance over other RNA structure prediction methods. For ncRNA benchmark with the redundant sequences removed, the performance evaluation is summarized in Additional file 1: Table S3 and REDfold can still have the best prediction accuracy (ACC=0.893). Furthermore, the RNA sequences with pseudoknot structure were taken from the ncRNA benchmark to assess the performance of structure prediction for RNAs with pseudoknots. Most RNA secondary structure prediction packages exclude pseudoknot structure due to extreme computational cost and it leads to accuracy degradation. However, REDfold can still have outstanding performance in terms of the accuracy metrics as illustrated in Table 5.

To further evaluate the prediction performance for the novel ncRNAs not present in the benchmark, RNA families with more than 100 members but excluded in the ncRNA benchmark were taken from the *Rfam* database for further testing. There are overall 10 RNA families and 1086 sequences, and the composition of the testing family groups is listed in Additional file 1: Table S2. Table 6 summarizes the prediction performance with respect to the structure prediction methods. As the deep learning model was trained to learn the structures of RNA families in the benchmark, the prediction of REDfold for the brand-new family is not as accurate as the learned RNA families. SPOTRNA uses ensemble learning that combines the predictions of multiple learning network models and hence obtains better generalization performance for the new family [17]. However, the prediction accuracy of REDfold can still be high among these prediction methods. Besides, REDfold is able to learn some new RNA structures from the features of RNAs in the benchmarks. For the new RNA families of SCV SLIV and ssNA-helicase RNA, the predictions of REDfold are accurate with ACC 0.916 and 0.906 respectively.

**Table 4:** Performance evaluation results based on the ncRNA benchmark

	ACC	SEN	PPV	F-Score	Log$_{10}$(Time)
REDfold	**0.950**	**0.952**	**0.939**	**0.946**	3.740
*RNAfold*	0.561	0.658	0.518	0.580	**2.772**
*RNAstructure*	0.555	0.646	0.513	0.572	3.636
*Probknot*	0.560	0.656	0.518	0.579	3.922
*CONTRAfold*	0.614	0.684	0.563	0.618	3.161
*SPOTRNA*	0.582	0.560	0.636	0.596	5.688
*Ufold*	0.904	0.936	0.865	0.900	5.212
*E2Efold*	0.416	0.295	0.342	0.317	5.189
*MXfold2*	0.712	0.707	0.677	0.692	4.325

Top-performing items are emphasized with bold font

**Table 5:** Performance evaluation results for the RNAs with pseudoknots based on the ncRNA benchmark

	ACC	SEN	PPV	F-Score	Log$_{10}$(Time)
REDfold	**0.923**	**0.936**	**0.912**	**0.924**	3.060
*RNAfold*	0.414	0.505	0.431	0.465	**2.182**
*RNAstructure*	0.414	0.500	0.431	0.463	3.459
*Probknot*	0.434	0.521	0.448	0.482	3.799
*CONTRAfold*	0.472	0.526	0.448	0.482	2.883
*SPOTRNA*	0.603	0.554	0.618	0.585	5.204
*Ufold*	0.852	0.905	0.820	0.861	4.650
*E2Efold*	0.466	0.415	0.467	0.440	4.646
*MXfold2*	0.623	0.577	0.639	0.607	3.799

Top-performing items are emphasized with bold font

**Table 6:** Performance evaluation results for the RNAs outside the ncRNA benchmark

	ACC	SEN	PPV	F-Score	Log$_{10}$(Time)
REDfold	0.654	0.519	0.702	0.597	2.064
*RNAfold*	0.615	0.711	0.592	0.646	**1.042**
*RNAstructure*	0.613	0.702	0.591	0.642	2.217
*Probknot*	0.638	**0.727**	0.613	0.665	2.547
*CONTRAfold*	0.663	0.715	0.643	0.677	1.663
*SPOTRNA*	**0.706**	0.671	**0.716**	**0.692**	4.197
*Ufold*	0.590	0.496	0.604	0.544	3.692
*E2Efold*	0.198	0.039	0.056	0.046	3.653
*MXfold2*	0.645	0.616	0.648	0.632	2.753

Top-performing items are emphasized with bold font

## Conclusions

Predicting RNA secondary structure is a challenging problem in computational biology. Various methods have been developed and the prediction approach based on thermodynamic models has been popular. As deep learning approaches have advanced substantially in terms of performance, the RNA secondary structure prediction based on DNNs can be more accurate. In this paper, we proposed REDfold, a novel algorithm for RNA secondary structure prediction based on a residual encoder-decoder learning network. REDfold incorporates *Resnet* with *FC-DenseNet* to make the learning model more efficient and effective for RNA structure prediction. Furthermore, it utilizes constrained optimization rather than dynamic programming to find the optimal structure, and hence the predicted structure is not restricted to nested folding structures. The comprehensive performance evaluation based on *RNAStralign* and ncRNA benchmark constructed from RNA families in the *Rfam* database shows that the proposed REDfold method outperforms popular RNA structure prediction methods in terms of prediction accuracy. The high accuracy of the REDfold makes the predicted structure close to the native structure. Besides, the REDfold algorithm can efficiently and accurately predict RNA structures with pseudoknots. Though the prediction based on the deep learning approach needs a large amount of training dataset, the prediction accuracy is better than traditional predictions. For the new RNA families, REDfold can still learn important features from the training dataset and have accurate predictions for some new RNA structures. As more and more ncRNAs are discovered, REDfold is capable of learning more critical features from these RNAs and making better structure predictions for exploring the new RNAs. Furthermore, REDfold is also computationally efficient that could be a useful tool for large-scale RNA analysis and synthesis.

## Supplementary Information

**Additional file 1**. **Appendix:** Tables for RNA Family Groups and Further Performance Evaluation of ncRNA Benchmark.

## Data Availability

The datasets analyzed and the source code for REDfold in this paper are available at https://github.com/aky3100/REDfold. The REDfold web server is freely available at https://redfold.ee.ncyu.edu.tw.

## Abbreviations

RNA: Ribonucleic acid
ncRNA: Noncoding RNA
circRNA: Circular RNA
BCM: Basic convolution module
CNN: Convolutional neural network
DCM: Dense connected module
DNN: Deep neural network
DP: Dynamic programming
LSTM: Long short term memory
ReLU: Rectified linear unit
