# Supplementary material for: REDfold: accurate RNA secondary structure prediction using residual encoder-decoder network
Source: BMC Bioinformatics. 2023 Mar 28;24:122. doi: 10.1186/s12859-023-05238-8 (PMC10044938; doi:10.1186/s12859-023-05238-8)
Supplement: Supplementary file 1 — Additional file 1. Appendix: Tables for RNA Family Groups and Further Performance Evaluation of ncRNA Benchmark. [file 12859_2023_5238_MOESM1_ESM.pdf]

Appendix: Tables for RNA Family Groups and Further Performance Evaluation of ncRNA Benchmark.

Table S1. List of RNA family groups and the number of sequences in the ncRNA benchmark. The families are grouped according the Rfam ID in the *Rfam* database 14.6.

RNA family group	Number of sequences	RNA family group	Number of sequences
RP L31 leader	2784	DUF RNA	2681
Twister ribozyme	1827	RAGATH RNA	1560
pemK RNA	1538	skipping-rope RNA	1423
S15-RNA	1167	Intron RNA	1144
SAM-RNA	880	RT-RNA	710
5S rRNA	691	glnA-RNA	647
Cyclic di-GMP riboswitch	624	S4-RNA	620
tRNA	584	IMES RNA	578
Downstream-peptide RNA	569	RP EL15 leader	536
algC RNA	492	raiA RNA	485
GA-cis RNA	481	chrB RNA	475
C4-2 RNA	469	tmRNA	466
drum RNA	463	RP L19 leader	459
RNase P class A	450	RP L2 leader	449
EFASI RNA	448	PhotoRC RNA	444
SpF59 sRNA	410	RP L17 leader	408
miRNA	404	NMT1 RNA	382
RP L25 leader	381	RP L4 leader	376
RP L13 leader	370	LOOT RNA	368
Transposase RNA	326	malK RNA	320
Mu-gpT-DE RNA	314	Clostridiales RNA	313
narK RNA	305	ROOL RNA	289
nhaA-I RNA	281	IsrR	279
6A RNA	273	crcB RNA	263
SRP RNA	261	Cobalamin Riboswitch RNA	260
IS605-orfB-I RNA	259	Rhodo-rpoB RNA	259
ivy-DE RNA	245	Actinomyces-1 RNA	234
Cyano-1 RNA	226	GP20 RNA	210

U2 snRNA	206
uup RNA	203
RP S10 Leader	199
nadA RNA	182
U4 spliceosomal RNA	174
FuFi-1 RNA	169
manA RNA	165
Moco RNA	159
engA RNA	154
Entero 5 CRE	153
6S RNA	147
AdoCbl variant RNA	145
M-box riboswitch	142
potC RNA	136
lysM-TM7 RNA	129
RP S16 leader	129
abiF RNA	126
cyVA-1 RNA	122

Flavobacterium-1 RNA	205
Freshwater-2 RNA	199
U6 spliceosomal RNA	187
U5 spliceosomal RNA	179
ZMP/ZTP Riboswitch	173
int-alpA RNA	167
TwoAYGGAY RNA	162
Cyclic di-GMP-I riboswitch	155
Hatchet ribozyme	153
leuA-Halobacteria RNA	152
Bacilli-1 RNA	145
FMN riboswitch	144
dfrA-dnaX RNA	138
Peptidase-S11 RNA	135
terC RNA	129
DABA-DC-AT RNA	126
HIV-RF RNA	123

Table S2. List of RNA family groups and the number of sequences for testing ncRNAs outside the benchmark. The families are grouped according the Rfam ID in the *Rfam* database 14.6.

RNA family group	Number of sequences
RP L20 leader	116
RNase P class B	113
RT-10 RNA	113
Twister-sister ribozyme	112
HCV SLIV	109
gltS RNA	108
DUF3268 RNA	106
tRNA-SeC	105
ssNA-helicase RNA	104
RP S15 leader	100

Table S3. Performance evaluation results based on the ncRNA benchmark with the redundant sequences removed.

	ACC	SEN	PPV	F-Score	Log_{10}(Time)
REDfold	**0.893**	**0.893**	**0.880**	**0.887**	2.513
RNAfold	0.532	0.639	0.501	0.562	**1.940**
RNAstructure	0.527	0.629	0.497	0.555	3.154
Probknot	0.540	0.644	0.508	0.568	3.488
CONTRAfold	0.583	0.665	0.546	0.600	2.616
SPOTRNA	0.663	0.654	0.637	0.645	4.913
Ufold	0.828	0.871	0.787	0.827	4.303
E2Efold	0.284	0.161	0.190	0.175	4.311
MXfold2	0.638	0.640	0.609	0.624	3.700
